# Reduction in Left Ventricular Ejection Fraction is Associated with Subsequent Cardiac Events in Outpatients with Chronic Heart Failure

**DOI:** 10.1038/s41598-019-53697-y

**Published:** 2019-11-21

**Authors:** Yoshitaka Okuhara, Masanori Asakura, Yoshiyuki Orihara, Daisuke Morisawa, Yuki Matsumoto, Yoshiro Naito, Takeshi Tsujino, Masaharu Ishihara, Tohru Masuyama

**Affiliations:** 1 Cardiovascular Division, Department of Internal Medicine, Hyogo College of Medicine, Hyogo, Japan; 2 Department of Pharmacy, School of Pharmacy, Hyogo University of Health Sciences, Kobe, Japan

**Keywords:** Heart failure, Heart failure

## Abstract

Left ventricular ejection fraction (LVEF) is critical for determining the prognosis and treatment of patients with heart failure (HF). However, the influence of serial LVEF changes in patients with stable chronic HF (CHF) has not yet been completely investigated. We analyzed data of 263 outpatients with CHF from the J-MELODIC study cohort and evaluated the frequency of cardiac events. We stratified patients into tertiles based on the relative difference in LVEF in 1 year and that at baseline. We found a significant difference in the cardiac event rate among the three groups (log-rank test, p = 0.042). We identified a relative 11% LVEF reduction as the optimal cutoff value based on the receiver operating characteristics analysis. LVEF (OR, 1.04; 95% CI, 1.01–1.07; p = 0.015) and E/e′ (OR, 1.06; 95% CI, 1.01–1.12; p = 0.023) at baseline were predictors of >11% LVEF reduction. After adjusting the variables including age and sex, >11% LVEF reduction was an independent predictor of subsequent cardiac events (HR, 5.79; 95% CI, 2.49–13.2; p < 0.001). In conclusion, patients with 1-year relative >11% LVEF reduction may have subsequent worsening outcomes. Such patients should be carefully followed-up as high risk population for development of cardiac events.

## Introduction

Left ventricular ejection fraction (LVEF) influences the diagnosis, prognosis, and management of patients with heart failure (HF). The LVEF is often used as a selection criterion in HF clinical trials. Recently, the European Society of Cardiology (ESC) guidelines proposed a new category for HF based on LVEF known as HF with reduced ejection fraction (HFrEF), HF with mid-range ejection fraction (HFmrEF), and HF with preserved ejection fraction (HFpEF), all of which are characterized by different pathologies^1,2^. However, LVEF gradually varies in several clinical settings and during long-term HF follow-up periods^3^.

An LVEF category change in serial LVEF measurements is reportedly an important prognostic factor in CHART-2 and SwedeHF registries^4,5^. An increase in LVEF correlates with improved long-term prognosis in patients with dilated cardiomyopathy^6^. Conversely, LVEF reduction is an important indicator of poor outcomes in those with drug-induced cardiomyopathy^7,8^. Therefore, it is important to manage patients with HF by focusing on LVEF changes. However, the predictors and cutoff point in LVEF change remain unclear in patients with chronic HF (CHF). In the present study, we aimed to evaluate the effects of 1-year LVEF change in outpatients with stable CHF.

## Material and Methods

### Study population

We retrospectively analyzed data from the Japanese multicenter J-MELODIC study to evaluate the effects of LVEF change in outpatients with CHF. The details of the J-MELODIC study design have previously been described^9,10^. Briefly, the study population comprised 320 outpatients with New York Heart Association (NYHA) II or III CHF and who received standard therapy including diuretics, renin–angiotensin–aldosterone system inhibitors (RAASi), and β-blockers (BB). The study included patients with any LVEF. LVEFs were measured according to the Simpson or Teichholz method, as appropriate. The primary endpoint was cardiovascular death or unplanned hospital admission for acute decompensated HF. This study was approved by the ethics committee of the Hyogo College of Medicine (No. 298) and was conducted in accordance with the Declaration of Helsinki; all patients provided written informed consents before inclusion in the study. We conducted all methods in accordance with the relevant guidelines and regulations.

### Study design

We excluded patients if they were lost to the 1-year follow-up period after the beginning of the study (n = 26); moreover, patients hospitalized for HF, those who died of any cause within 1 year after the beginning of the study (n = 26), and those with missing LVEF data (n = 5) required to examine the effects of 1-year LVEF changes were excluded. Consequently, we evaluated data of 263 outpatients with CHF from the J-MELODIC study. None of the patients underwent cardiac surgery, percutaneous coronary interventions, or implantable device surgery (pacemaker/implantable cardioverter defibrillator/cardiac resynchronization therapy) during the 1-year follow-up period. As shown in Supplementary Table, data from patients hospitalized for HF or those who died of any cause during the 1-year follow-up period (n = 26) were compared with data from patients included in the study (n = 263). As a result, patients with 1-year events exhibited higher NYHA class, blood urea nitrogen, brain natriuretic peptide (BNP) levels, and E/e′ values as well as lower body mass index (BMI), systolic blood pressure (SBP), and estimated glomerular filtration rates (eGFR) levels compared with those without 1-year events. We evaluated cardiac events as a composite endpoint of cardiovascular death or unplanned hospital admission for acute decompensated HF. We defined 1-year LVEF change (ΔLVEF) as the relative difference in LVEF in 1 year and that at baseline [(1year LVEF – baseline LVEF)/baseline LVEF]. We stratified the eligible patients into three groups according to LVEF change tertiles: lowest tertile group (ΔLVEF < −2.2%, n = 88), middle tertile group (−2.2% ≤ ΔLVEF < 5.6%, n = 88), and highest tertile group (ΔLVEF ≥ 5.6%, n = 87) to evaluate the association between LVEF change and the cardiac event rate. We defined the optimal cutoff point of LVEF change for predicting subsequent cardiac events as the point on the receiver operating characteristic (ROC) curve maximizing the Youden index. We defined HFpEF as HF with LVEF ≥50%; HFmrEF as that with LVEF 40%–49%; and HFrEF as that with LVEF <40% according to the 2016 ESC guidelines^1^.

### Statistical analysis

In our study, we expressed continuous variables as mean ± standard deviation (SD) if they fit a normal distribution and presented skewed values as medians and interquartile range (IQR). We investigated variables fitting a normal distribution using paired/unpaired *t*-tests or ANOVA. We used Wilcoxon signed-rank/Mann–Whitney *U* or Kruskal–Wallis tests when variables did not fit a normal distribution. We examined categorical variables using chi-square test, Fisher’s exact test, or McNemar test. We used an ROC curve analysis to assess the diagnostic performance of the 1-year LVEF change for predicting subsequent cardiac events, with the area under the curve (AUC) calculated accordingly. We defined the optimal cutoff value based on the maximum Youden index, calculated as “sensitivity + specificity − 1.” We used the Spearman rank correlation coefficient to determine the association between baseline LVEF, change in left ventricular diastolic diameter (LVDd), and 1-year LVEF change. We also performed univariable and multivariable analyses to identify factors predicting 1-year LVEF change and included variables with *p* ≤ 0.1 in univariable analysis into the multivariable analysis. We performed the subsequent event analysis using the Kaplan–Meier analysis and comparisons using the log-rank test. Multivariable Cox proportional hazards regression analysis was performed to estimate hazard ratios (HRs) for subsequent cardiac events with adjustment for variables including age and sex. We reported the results as odds ratios (ORs) or HRs with 95% confidence intervals (95% CIs). A *p* value of <0.05 was considered as statistically significant, and all statistical analyses were performed using JMP software, version 11.2 (SAS Institute, Cary, NC, USA).

## Results

### Patient population

**Figure 1:**
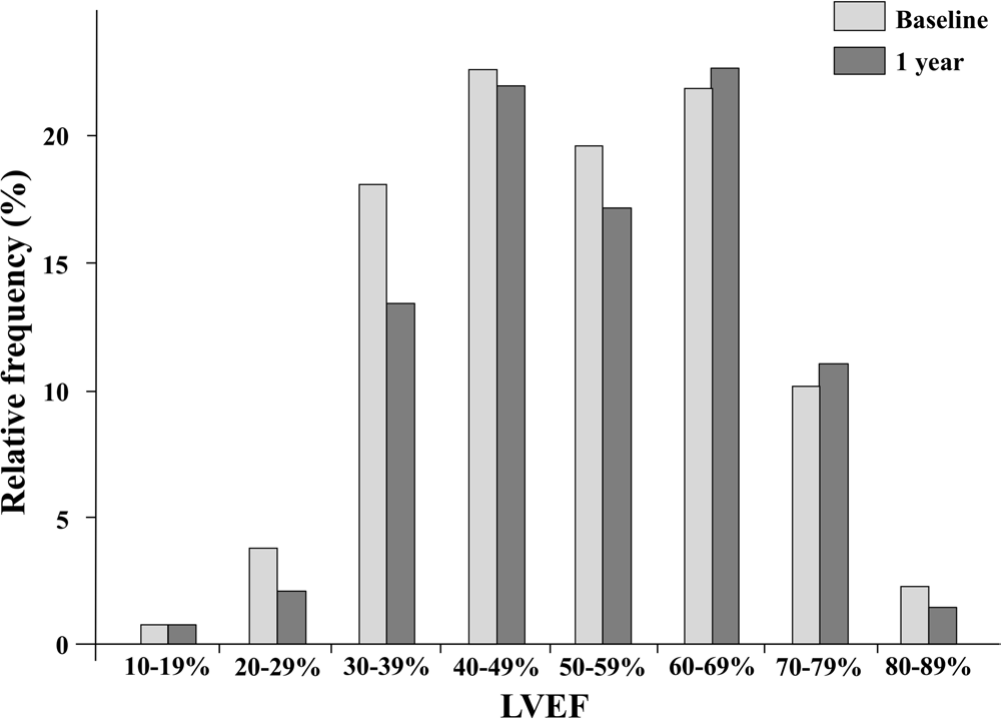
Histogram of left ventricular ejection fraction (LVEF) at baseline and at 1 year.

**Figure 2:**
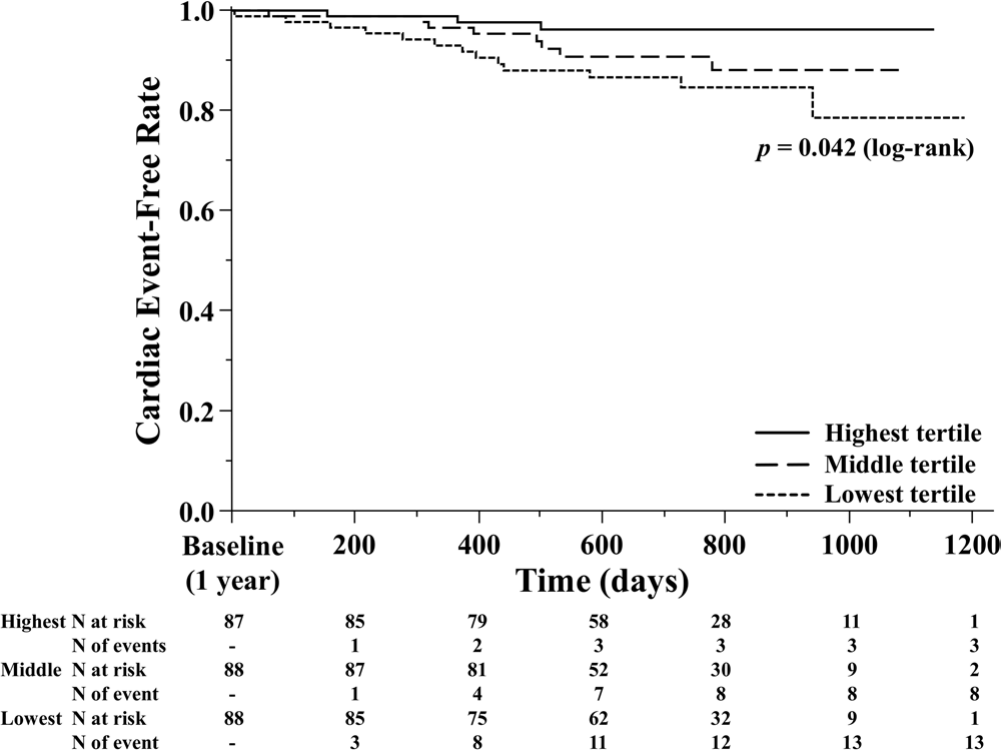
Kaplan–Meier survival curves for cardiovascular death or unplanned hospital admission according to tertiles of 1-year change in left ventricular ejection fraction (LVEF). Lowest tertile group (ΔLVEF < −2.2%, n = 88); Middle tertile group (−2.2% ≤ ΔLVEF < 5.6%, n = 88); Highest tertile group (ΔLVEF ≥ 5.6%, n = 87).

**Figure 3:**
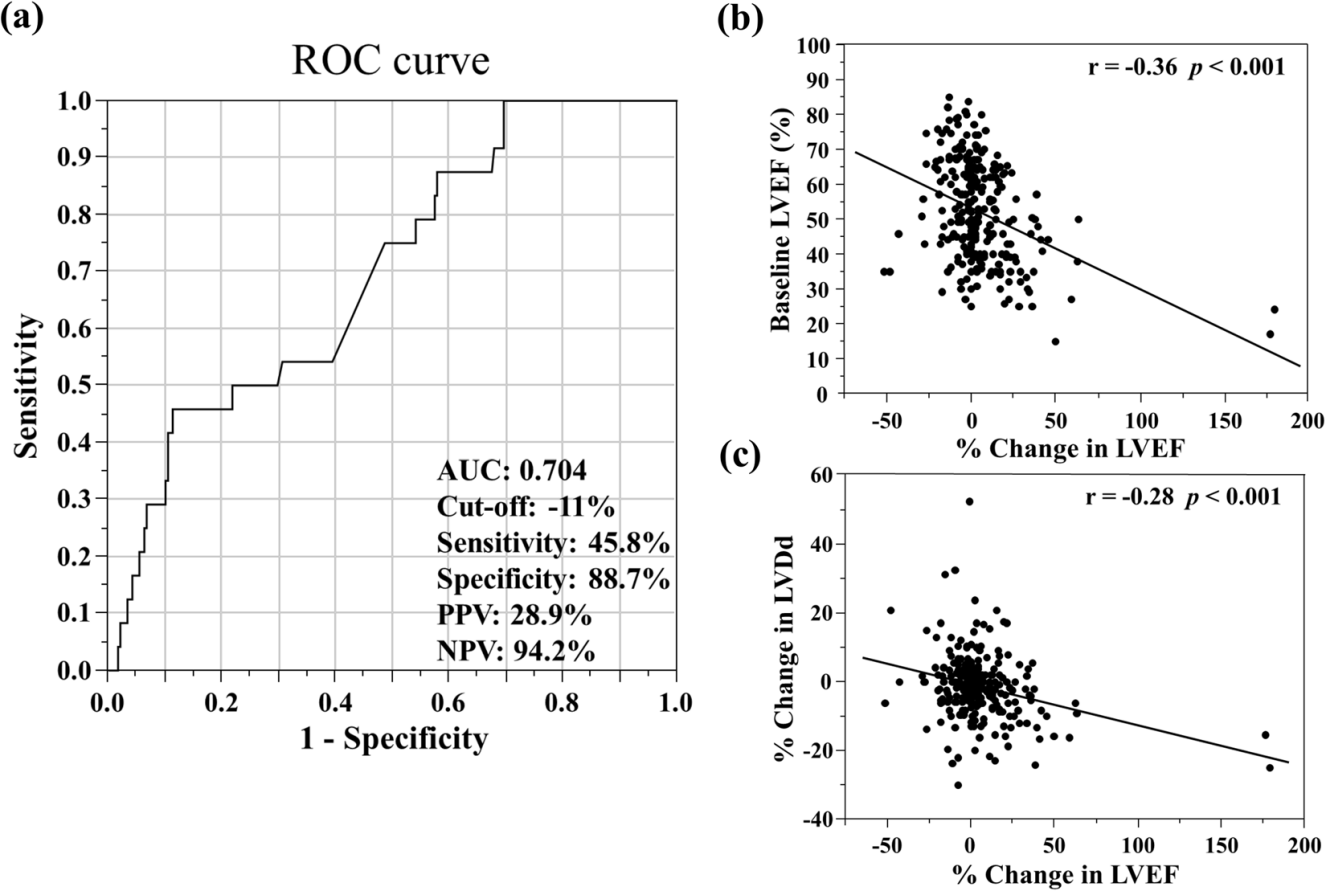
(**a**) Receiver operating characteristic (ROC) analysis of left ventricular ejection fraction (LVEF) change in 1 year for the composite outcome in patients with chronic heart failure. AUC, area under the curve. (**b**) Relationship between baseline LVEF and 1-year LVEF change. (**c**) Relationship between left ventricular diastolic diameter (LVDd) change and 1-year LVEF change.

**Figure 4:**
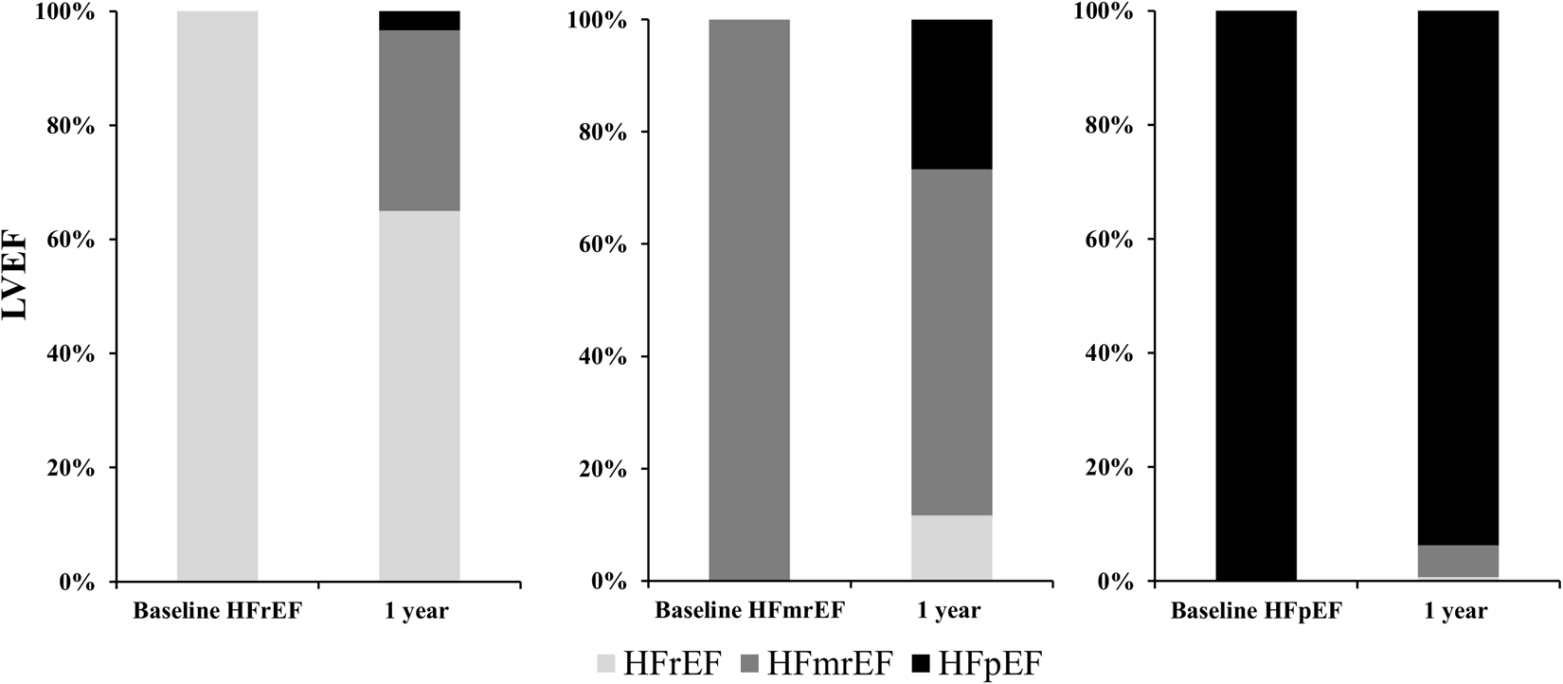
Changes in left ventricular ejection fraction (LVEF) category by ESC classification from baseline to 1-year follow-up. (Left) Heart failure with reduced ejection fraction (HFrEF); (Center) Heart failure with mid-range ejection fraction (HFmrEF); (Right) Heart failure with preserved ejection fraction (HFpEF).

Figure 1 shows a histogram of LVEF at baseline and at 1 year. Median LVEFs were 52% (IQR 40%–64%) at baseline and 53% (IQR 42%–64%) at 1 year. The baseline and 1-year characteristics were stratified by tertiles according to the relative LVEF change values (Table 1). In the lowest tertile group, the incidence of NYHA III CHF and HFpEF was high. The incidence of HFrEF and dilated cardiomyopathy was low in the lowest tertile group. BNP and E/e′ levels were higher at 1 year in the lowest tertile group (p = 0.069 and 0.113, respectively). HF types significantly changed in the lowest and highest tertile groups. The eGFR and BNP values were significantly decreased at 1 year compared with those at baseline in the highest tertile group only. We found no significant differences in terms of oral medications used among groups. Kaplan–Meier analysis showed a significant difference in the cardiac event rates among the three groups (log-rank, p = 0.042; Fig. 2). Figure 3a shows the ROC curve for relative LVEF change in 1 year to predict subsequent cardiac events. The optimal cutoff value based on the ROC curve was set at a relative 11% LVEF reduction corresponding to 45.8% sensitivity, 88.7% specificity, 28.9% positive predictive value (PPV), 94.2% negative predictive value (NPV), and an AUC of 0.704 (95% CI = 0.596–0.804). Relative LVEF reduction of >11% was observed in 14.4% of the 263 patients. As shown in Table 2, at baseline, patients with >11% LVEF reduction showed significantly lower hemoglobin levels and higher BNP levels, LVEFs, and E/e′ values than those without >11% LVEF reduction. Moreover, at the 1-year follow-up, patients with >11% LVEF reduction exhibited significantly higher edema rates and lower hemoglobin levels and LVEF than those without >11% LVEF reduction. Furthermore, patients with >11% LVEF reduction showed higher 1-year E/e′ values than those without >11% LVEF reduction (p = 0.066). Patients without >11% LVEF reduction showed lower edema rates; lower eGFR, BNP, and LVDd levels; and higher LVEF at the 1-year follow-up than at baseline. HF type significantly changed between both patient groups. As shown in Fig. 3b,c, the 1-year LVEF change was inversely correlated with baseline LVEF (r = −0.36, *p* < 0.001) and change in LVDd in 1 year (r = −0.28, *p* < 0.001). The category changes based on the ESC HF classification are shown in Fig. 4. Patients with HFrEF at baseline were distributed across the HFrEF (65%), HFmrEF (32%), and HFpEF (3%) categories after the 1-year follow-up period. Overall, 61% patients with HFmrEF at baseline remained in the HFmrEF category, and 12% and 27% of those shifted to HFrEF and HFpEF, respectively. Most patients with HFpEF at baseline remained in the HFpEF (94%) category, and 1% and 5% of those shifted to HFrEF and HFmrEF, respectively.

**Table 1:** Patient characteristics in different 1-year LVEF change tertiles.

	Lowest tertile group (n = 88)		Middle tertile group (n = 88)		Highest tertile group (n = 87)	
	Baseline	1-year	Baseline	1-year	Baseline	1-year
Age, median (IQR), y	74 (66–79)		73 (64–78)		71 (64–75)	
Male, n (%)	53 (60.2)		57 (64.8)		51 (58.6)	
NYHA class, n (%)						
I		11 (12.5)		16 (18.2)		18 (20.7)
II	76 (86.4)	68 (77.3)	82 (93.2)	66 (75.0)	84 (96.6)	66 (75.9)
III	12 (13.6)*	8 (9.1)	6 (6.8)	6 (6.8)	3 (3.5)	3 (3.5)
IV		1 (1.1)		0 (0)		0 (0)
Edema, n (%)	34 (38.6)*	28 (31.8)	19 (21.6)	16 (18.2)	30 (34.5)	25 (28.7)
BMI, mean (SD), kg/m2	23.3 (3.8)	23.2 (4.0)	23.4 (3.7)	23.6 (3.8)	23.6 (4.0)	23.7 (3.9)
SBP, median (IQR), mmHg	128 (116–138)	126 (110–139)	126 (116–135)	125 (114–138)	130 (114–140)	122 (115–135)
HR, mean (SD), bpm	73 (13)	72 (12)	70 (14)	71 (11)	72 (14)	71 (11)
Hemoglobin, mean (SD), g/dL	12.6 (2.0)	12.7 (2.2)	13.1 (1.8)	13.2 (1.8)	12.8 (1.7)	12.7 (1.9)
BUN, median (IQR), mg/dL	21.0 (16.0–23.8)	18.6 (15.0–23.0)	20.6 (16.0–26.0)	21.0 (15.0–26.0)	19.0 (15.0–23.0)	18.2 (14.0–25.0)
eGFR, median (IQR), mL/min/1.73 m2	57.5 (43.8–68.6)	55.9 (42.0–71.4)	51.6 (41.2–61.2)	50.2 (39.0–64.9)	56.7 (43.4–69.8)	49.8 (42.9–71.7)^†^
BNP, median (IQR), pg/mL	121.5 (52.6–235)	112 (50.0–186.2)	111 (38.8–270.3)	90.0 (37.7–184.5)	89.7 (39.7–193.4)	67.0 (33.6–152)^†^
LVEF, median (IQR), %	57 (45–70)	51 (40–62)^†^	53 (42–64)	53 (42–63)^†^	46 (35–59)	58 (44–69)^†^
**HF type**, **n (%)**						
HFrEF	13 (14.8)*	21 (23.9)^†^	17 (19.3)	15 (17.1)	30 (34.5)	11 (12.6)^†^
HFmrEF	20 (22.7)	21 (23.9)	20 (22.7)	21 (23.9)	20 (23.0)	22 (25.3)
HFpEF	55 (62.5)*	46 (52.2)^†^	51 (58.0)	52 (59.0)	37 (42.5)	54 (62.1)^†^
LVDd, median (IQR), mm	53 (47–59)	52 (48–59)	54 (48–59)	53 (47–59)	53 (49–58)	51 (47–56)^†^
E/e′, median (IQR)	12.5 (9.6–16.0)	12.1 (8.6–17.4)	11.9 (8.1–15.8)	11.5 (8.4–15.5)	11.5 (8.8–15.1)	10.2 (8.6–14.2)
**Etiology**, **n (%)**						
Ischemic etiology	29 (33.0)		37 (42.1)		23 (26.4)	
DCM	12 (13.6)*		19 (21.6)		28 (32.2)	
AF or AFL	39 (44.3)		32 (36.4)		32 (36.8)	
Pacemaker implantation	5 (5.7)		4 (4.6)		8 (9.2)	
Hypertension, n (%)	55 (62.5)		55 (62.5)		58 (66.7)	
Diabetes mellitus, n (%)	26 (29.6)*		37 (42.1)		19 (21.8)	
**Oral medications**, **n (%)**						
Loop diuretics	88 (100)		88 (100)		87 (100)	
Furosemide equivalent, median (IQR), mg	20 (20–40)		20 (20–40)		20 (20–40)	
Aldosterone antagonists	34 (38.6)		41 (46.6)		34 (39.1)	
ACE-inhibitors or ARBs	61 (69.3)		66 (75.0)		64 (73.6)	
β-blockers	40 (45.5)		52 (59.1)		47 (54.0)	

LVEF, left ventricular ejection fraction; Lowest tertile group, ΔLVEF < −2.2%; Middle tertile group, −2.2% ≤ ΔLVEF < 5.6%; Highest tertile group, ΔLVEF ≥ 5.6%; IQR, interquartile range; SD, standard deviation; NYHA, New York Heart Association; BMI, body mass index; SBP, systolic blood pressure; HR, heart rate; BUN, blood urea nitrogen; eGFR, estimated glomerular filtration rate; BNP, brain natriuretic peptide; HF, heart failure; HFrEF, heart failure with reduced ejection fraction; HFmrEF, heart failure with mid-range ejection fraction; HFpEF, heart failure with preserved ejection fraction; LVDd, left ventricular diastolic dimension; DCM, dilated cardiomyopathy; AF, atrial fibrillation; AFL, atrial flutter; ACE, angiotensin-converting enzyme; ARB, angiotensin receptor blocker. *p < 0.05 vs. same period among tertiles, ^†^*p* < 0.05 vs. baseline.

**Table 2:** Characteristics of patients by relative 11% LVEF reduction in 1 year.

	Patients with LVEF reduction (n = 38)		Patients without LVEF reduction (n = 225)	
	Baseline	1-year	Baseline	1-year
Age, median (IQR), y	74 (66–80)		72 (65–77)	
Male, n (%)	19 (50.0)		142 (63.1)	
**NYHA class**, **n (%)**				
I		5 (13.2)		40 (17.8)
II	32 (84.2)	28 (73.7)	210 (93.3)	172 (76.4)
III	6 (15.8)	4 (10.5)	15 (6.7)	13 (5.8)
IV		1 (2.6)		0 (0.0)
Edema, n (%)	16 (42.1)	17 (44.7)*	67 (29.8)	52 (23.1)^†^
BMI, median (IQR), kg/m2	22.1 (19.2–25.8)	22.4 (19.1–25.4)	23.5 (21.0–26.0)	23.6 (20.9–25.9)
SBP, mean (SD), mmHg	127 (18)	124 (19)	126 (16)	125 (18)
HR, median (IQR), bpm	72 (64–81)	72 (66–83)	70 (62–79)	71 (62–78)
Hemoglobin, mean (SD), g/dL	12.2 (2.0)*	12.2 (2.1)*	12.9 (1.8)	13.0 (1.9)
BUN, median (IQR), mg/dL	22 (17–23)	18 (15–24)	20 (16–25)	19 (15–25)
eGFR, median (IQR), mL/min/1.73 m^2^	53.8 (41.4–68.1)	52.5 (36.9–71.5)	55.3 (43.1–65.3)	51.9 (42.0–68.0)^†^
BNP, median (IQR), pg/mL	161 (56.3–327.3)*	136 (51.7–239.8)	100 (42.6–201)	76.6 (39.0–153.4)^†^
LVEF, median (IQR), %	58 (15)*	47 (14)*†	51 (14)	55 (14)^†^
**HF type**, **n (%)**				
HFrEF	5 (13.2)	11 (29.0)^†^	55 (24.4)	36 (16.0)^†^
HFmrEF	9 (23.7)	8 (21.1)	51 (22.7)	56 (24.9)
HFpEF	24 (63.2)	19 (50.0)^†^	119 (52.9)	133 (59.1)^†^
LVDd, median (IQR), mm	52.6 (47.0–58.3)	51.7 (45.8–59.0)	53.8 (48.9–59.0)	52.0 (47.0–58.0)^†^
E/e′, median (IQR)	13.5 (10.9–18.2)*	13.2 (9.0–25.7)	11.7 (8.5–15.6)	10.9 (8.4–15.0)
**Etiology**, **n (%)**				
Ischemic etiology	11 (29.0)		78 (34.7)	
DCM	7 (18.4)		52 (23.1)	
AF or AFL	19 (50.0)		84 (37.3)	
Pacemaker implantation	3 (7.9)		14 (6.2)	
Hypertension, n (%)	21 (55.3)		147 (65.3)	
Diabetes mellitus, n (%)	9 (23.7)		73 (32.4)	
**Oral medications**, **n (%)**				
Loop diuretics	38 (100)		225 (100)	
Furosemide equivalent, median (IQR), mg	20 (20–40)		20 (20–40)	
Aldosterone antagonists	16 (42.1)		93 (41.3)	
ACE-inhibitors or ARBs	28 (73.7)		163 (72.4)	
β-blockers	20 (52.6)		119 (52.9)	

LVEF, left ventricular ejection fraction; IQR, interquartile range; SD, standard deviation; NYHA, New York Heart Association; BMI, body mass index; SBP, systolic blood pressure; HR, heart rate; BUN, blood urea nitrogen; eGFR, estimated glomerular filtration rate; BNP, brain natriuretic peptide; HF, heart failure; HFrEF, heart failure with reduced ejection fraction; HFmrEF, heart failure with mid-range ejection fraction; HFpEF, heart failure with preserved ejection fraction; LVDd, left ventricular diastolic dimension; DCM, dilated cardiomyopathy; AF, atrial fibrillation; AFL, atrial flutter; ACE, angiotensin-converting enzyme; ARB, angiotensin receptor blocker. ^*^p < 0.05 vs. same period in patients without LVEF reduction, ^†^*p* < 0.05 vs. baseline.

### Predictors of 1-year LVEF reduction

The results of multivariable analysis incorporating significant risk factors from univariable analyses revealed that baseline LVEF (OR = 1.04; 95% CI = 1.01–1.07; *p* = 0.015) and E/e′ values (OR = 1.06; 95% CI = 1.01–1.12; *p* = 0.023) were predictors of the 1-year > 11% LVEF reduction (Table 3).

**Table 3:** Univariable and multivariable analysis of factors related to relative >11% LVEF reduction.

	Univariable analysis			Multivariable analysis		
	Odds ratio (95% CI)		*p* value	Odds ratio (95% CI)		*p* value
Age	1.02	0.98–1.05	0.367			
Male	0.58	0.29–1.17	0.128			
BMI	0.93	0.85–1.03	0.160			
SBP	1.00	0.98–1.03	0.710			
HR	1.02	0.99–1.05	0.078	1.03	0.99–1.06	0.083
Hb	0.81	0.67–0.98	0.033	0.94	0.74–1.18	0.597
eGFR	1.00	0.98–1.02	0.718			
Log BNP	1.17	0.63–2.22	0.624			
LVEF	1.03	1.01–1.06	0.010	1.04	1.01–1.07	0.015
LVDd	0.98	0.94–1.02	0.340			
E/e′	1.06	1.01–1.11	0.013	1.06	1.01–1.12	0.023
Diabetes mellitus	0.65	0.28–1.39	0.271			
Ischemia etiology	0.77	0.35–1.59	0.486			
AF or AFL	1.68	0.84–3.37	0.143			
Aldosterone antagonists	1.03	0.81–2.06	0.929			
ACE-inhibitors or ARBs	1.07	0.50–2.42	0.874			
β-blockers	0.99	0.50–1.98	0.977			

LVEF, left ventricular ejection fraction; CI, confidence intervals; BMI, body mass index; SBP, systolic blood pressure; HR, heart rate; Hb, hemoglobin; eGFR, estimated glomerular filtration rate; BNP, brain natriuretic peptide; LVDd, left ventricular diastolic diameter; AF, atrial fibrillation; AFL, atrial flutter; ACE, angiotensin-converting enzyme; ARB, angiotensin receptor blocker.

### LVEF reduction and subsequent outcome

**Figure 5:**
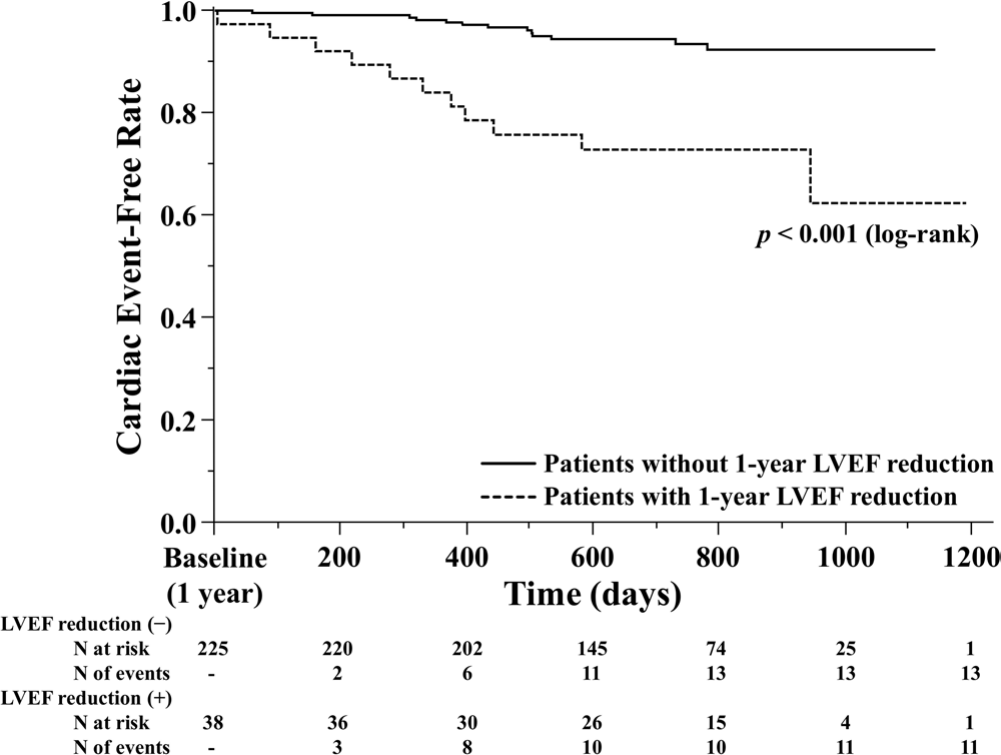
Kaplan–Meier curves for cardiovascular death or unplanned hospital admission according to 1-year relative 11% left ventricular ejection fraction (LVEF) reduction in patients with chronic heart failure.

Median follow-up period was 700 days (range, 497–860). Based on our Kaplan–Meier analysis stratified by tertiles, the cumulative cardiac event rates were 21.4% [cardiovascular death (n = 3), HF admission (n = 10)] in lowest tertile group, 11.9% [cardiovascular death (n = 1), HF admission (n = 7)] in the middle tertile group, and 3.8% [cardiovascular death (n = 0), HF admission (n = 3)] in the highest tertile group (Fig. 2). Based on the Kaplan–Meier analysis divided at the cutoff value of relative 11% LVEF reduction, the cumulative cardiac event rates were 7.6% [cardiovascular death (n = 2), HF admission (n = 11)] in patients without >11% LVEF reduction and 37.5% [cardiovascular death (n = 2), HF admission (n = 9)] in those with >11% LVEF reduction (log-rank, *p* < 0.001; Fig. 5). As shown in Table 4, our multivariable analysis revealed that after adjusting the variables including age and sex, >11% LVEF reduction was an independent predictor of subsequent cardiac events (HR, 5.79; 95% CI, 2.49–13.2; p < 0.001).

**Table 4:** Cox proportional hazard regression analysis of predictors of cardiac events in patients with chronic heart failure.

	Univariable analysis			Multivariable analysis		
	Hazard ratio (95% CI)		*p* value	Hazard ratio (95% CI)		*p* value
LVEF reduction	5.2	2.30–11.7	<0.001	5.79	2.49–13.2	<0.001
Age	1.05	1.00–1.10	0.032	1.05	1.01–1.10	0.029
Male	2.00	0.84–5.51	0.123	3.14	1.27–8.88	0.012
Diabetes mellitus	1.14	0.46–2.59	0.764.			
Ischemic etiology	0.53	0.18–1.32	0.184			
**At Baseline**						
BMI	0.94	0.84–1.04	0.248			
SBP	0.99	0.94–1.02	0.441			
Hb	0.96	0.78–1.19	0.727			
eGFR	0.98	0.96–1.01	0.194			
LVEF	0.99	0.96–1.02	0.457			
Log BNP	0.98	0.48–2.05	0.962			
**At 1 year**						
BMI	0.90	0.81–1.01	0.063			
SBP	1.00	0.98–1.02	0.940			
Hb	0.95	0.77–1.16	0.607			
eGFR	0.99	0.97–1.14	0.555			
LVEF	0.96	0.94–0.99	0.012			
Log BNP	17.10	5.94–51.5	<0.001			

CI, confidence intervals; LVEF, left ventricular ejection fraction; BMI, body mass index; SBP, systolic blood pressure; Hb, hemoglobin; eGFR, estimated glomerular filtration rate; LVEF, left ventricular ejection fraction; BNP, brain natriuretic peptide.

## Discussion

The major findings of this study are as follows: First, a 1-year LVEF change was associated with subsequent outcomes in outpatients with CHF with the cutoff value being a relative 11% LVEF reduction. Second, the predictors for a 1-year >11% LVEF reduction included the baseline LVEF and E/e′ values. Patients with >11% LVEF reduction showed higher baseline and 1-year E/e′ values than those without >11% LVEF reduction. Finally, the 1-year >11% LVEF reduction was an independent predictor after adjusting the variables including age and sex. Lasting high E/e′ could reduce LVEF in patients with CHF, which leads to subsequent worsening outcomes. CHF patients with >11% LVEF reduction should be carefully followed-up as high risk population for development of cardiac events.

LVEF changes occur gradually. A small prospective study reported that approximately one-third of patients with acute HF recovered their LVEF, whereas normalized LVEF was lost in 55% of the patients in the subsequent 2-year period^11,12^. In a 15-year follow-up study of HF investigating LVEF changes, the Loess curves of long-term LVEF trajectories showed an inverted U shape^3^. Particularly, LVEF substantially increased during the first year, maintained their level up to a decade, and gradually decreased thereafter. The study suggested that incomplete LVEF recovery during the first year and LVEF reduction in the chronic phase correlated with poor survival. Therefore, prognostic factors for LVEF change may vary between acute and chronic phases in patients with HF. In the present study including patients with stable CHF, LVEF varied during the 1-year follow-up period and the relative >11% LVEF reduction was significantly associated with subsequent worsening outcomes. Although the AUC was not sufficiently high, the NPV was high, suggesting that patients without >11% LVEF reduction could have less subsequent worsening outcomes compared with the other patients. LVEF reduction reportedly resulted in myocardial damage and poor prognoses in patients with chemo-induced cardiotoxicity as well as in patients with CHF during a long-term follow-up^3,5,8^. However, these studies included patients with acute HF and with inconstant intervals between the first and second LVEF measurements. In addition, the patients admitted due to HF during the measurement interval were included, which may have led to LVEF reductions and worsening outcomes. Our study included outpatients with CHF from the J-MELODIC study and excluded those with worsening events during echocardiographic assessments. Over 90% of the patients were able to maintain NYHA II at 1 year; the patients showed no significant change in SBP and HR at 1 year, which may affect LVEF measurements. These results indicated that our study examined outpatients with stable CHF.

Our study showed that the 1-year LVEF and BNP values were both significantly associated with subsequent cardiac events in patients with stable CHF. These results are consistent with those in studies that reported an inverse correlation between LVEF and worsening outcomes and showed that BNP is a strong prognostic factor for HF^13,14^. On the other hand, baseline BNP was not associated with subsequent cardiac events, suggesting that BNP change also should be considered when predicting prognosis. Not only LVEF but also BNP change over time, and sequential BNP reduction was reportedly associated with subsequent favorable prognosis in patients with CHF^15^. As a sub-analysis, we compared patients with BNP increase in 1 year and those with BNP reduction in 1 year. Consequently, patients with BNP reduction showed lower subsequent cardiac events rate than those with BNP increase (Supplementary Fig.). These results suggest that not only LVEF but also BNP levels are required to evaluate the changes. Moreover, SBP, hemoglobin, and eGFR values were not associated with subsequent cardiac events. These factors have previously been shown to be associated with poor outcomes in patients with HF. However, some registries have suggested that these factors were not always associated with poor outcomes, especially in the stable phase of HF^4,16,17^. As shown in the Supplementary Table, the patients who died due to any cause or were hospitalized for HF during the 1-year follow-up period showed worse status compared with that of others. Excluding such patients may have affected our results.

Neurohumoral activation causes the deterioration and progression of CHF. Sustained neurohumoral activation increases cardiac wall stress and reactive oxygen species generation, leading to ventricular dilation and HF progression^18^. In the present study, the LVEF change was inversely correlated with a 1-year LVDd change. LVEF reduction may reflect cardiac remodeling distinguished by increased fibrosis and abundance of collagen. These adverse reactions correlated with worsening outcomes in patients with HF. In addition, LVEF reduction was associated with increased level of serum high-sensitivity cardiac troponin T in anthracycline-induced cardiotoxicity^19^, thereby strongly supporting the notion that LVEF reduction worsens subsequent outcomes during the chronic phase.

Interestingly, in our study, a high baseline LVEF was associated to a 1-year LVEF reduction. Several studies have reported an inverse correlation between the baseline LVEF and LVEF changes, which is consistent with our results^20^. Patients with lower baseline LVEF reportedly exhibit a higher incidence of myocardial stunning and thus a greater likelihood of LVEF recovery following acute myocardial infarctions^21^. In contrast, high baseline LVEF was associated with subsequent LVEF reduction in patients with valve diseases^22^. Reasons for the inverse association between baseline LVEF and LVEF change have not yet been completely elucidated but might be explained by the statistical phenomenon of “regression toward the mean value”. Moreover, a high E/e′ value at baseline was a predictor of subsequent 1-year LVEF reduction. A study reported that an LVEF change was inversely correlated with the E/e′ value at baseline and that improvement in LVEF was attenuated when the E/e′ value was high. High baseline atrial pressure values significantly predicted subsequent LVEF reductions following mitral valve reconstructive surgery^22,23^. Moreover, eGFR was significantly decreased in patients without >11% LVEF reduction. Although decreased eGFR reflects a renal blood flow reduction, patients without LVEF reduction may achieve decongestion at the expense of kidney function as BNP and edema improved only in patients without LVEF reduction. Incomplete decongestion has reportedly been associated with subsequent poor prognoses. In contrast, complete decongestion induces improved subsequent prognoses even in cases wherein renal function is sacrificed^24^. Therefore, sustained decongestion at the expense of renal function may induce decreased wall stress, resulting in preserved LVEF. Thus, chronic increased cardiac wall stress leads to chamber dilatation and LVEF reduction.

LVEF changes have been examined according to the ESC classification in the Chronic Heart Failure Analysis and Registry in the Tohoku District-2 Study (CHART-2 Study), a multicenter, prospective observational study in Japan^4^. In that study, most patients with HFpEF remained in the same category over time and those with HFmrEF and HFrEF often moved to other categories. Similarly, in our study, most patients with HFpEF remained in the same category at the 1-year follow-up. Conversely, approximately 35% patients with HFrEF and 38% patients with HFmrEF moved to another category. These results suggest that an assessment of LVEF should consider previous LVEFs as well as the evaluation period in patients with HF. The pathophysiology between HFrEF and HFpEF is not considered homogenous. Although the efficacy of RAASi and BB has been established in patients with HFrEF, no evidence exists for the treatment of HFpEF, partly because clinical trials of HFpEF have included several patients with pseudo-HFpEF (those who were originally patients with HFrEF and HFmrEF but recovered their LVEF to ≥50%). Further studies are required to determine a true LVEF category in patients with HF.

### Study limitations

The major limitations of our study are its retrospective nature and the relatively small sample size. In addition, our observations may have been influenced by residual measured and/or unmeasured confounders, and the results may have been subjected to residual confounding that cannot be completely adjusted. Further, the echocardiographic data were reported by site, and no centralized analysis of LVEF and other echocardiographic data were performed in a core laboratory as these analyses occurred in different centers by various operators. The LVEFs were not uniformly measured and were not endpoints in the J-MELODIC registry. In addition, diastolic function and stroke volumes—the two essential variables—were not measured. Therefore, the interpretation of echocardiography data may be the most pronounced limitation in our study. Further, all the patients received furosemide or azosemide in the J-MELODIC registry. In addition, daily oral furosemide equivalent dose was very low compared with that in major HF registries. Although this study focused on patients with stable CHF, it is one of the limitations of this study. Moreover, 1-year LVEFs were compared with baseline values only, and detailed LVEF changes were not evaluated during the year. Moreover, unfortunately, the AUC for LVEF reduction was not sufficiently high. A more precise cutoff value should be established for each HF phase (acute or chronic) and category (HFpEF, HFmrEF, or HFrEF) in future studies. Finally, data regarding the details of oral medications at 1-year follow-up and dose adjustment of BB, RAASi, MRA, and diuretic doses were not reported in the J-MELDIC registry. Reportedly, BB and RAASi improve LVEF in patients with HFrEF. Moreover, withdrawal medication led to subsequent LVEF reduction and left ventricular dilatation with increased BP and HR in clinically stable patients with dilated cardiomyopathy^25^. Further study is needed to clarify the effects of LVEF change in patients with CHF.

## Conclusion

In conclusion, 1-year relative >11% LVEF reduction was a significant predictor of subsequent cardiac events, and baseline LVEF and E/e′ values predicted this 1-year >11% LVEF reduction.

## Supplementary information

Supplementary Information
