# Supplementary material for: Reduction in Left Ventricular Ejection Fraction is Associated with Subsequent Cardiac Events in Outpatients with Chronic Heart Failure
Source: Sci Rep. 2019 Nov 21;9:17271. doi: 10.1038/s41598-019-53697-y (PMC6872762; doi:10.1038/s41598-019-53697-y)

**Supplementary Information**

**Reduction in Left Ventricular Ejection Fraction is Associated with Subsequent Cardiac Events in Outpatients with Chronic Heart Failure**

Yoshitaka Okuhara, MD, PhD,^1^ Masanori Asakura, MD, PhD,^1^ Yoshiyuki Orihara, MD,^1^ Daisuke Morisawa, MD, PhD,^1^ Yuki Matsumoto MD,^1^ Yoshiro Naito, MD, PhD,^1^ Takeshi Tsujino, MD, PhD,^2^ Masaharu Ishihara, MD PhD,^1^ Tohru Masuyama, MD, PhD^1^

^1^Cardiovascular Division, Department of Internal Medicine, Hyogo College of Medicine, Hyogo, Japan

^2^Department of Pharmacy, School of Pharmacy, Hyogo University of Health Sciences, Kobe, Japan

Address for correspondence

Yoshitaka Okuhara, MD, PhD

Cardiovascular Division, Department of Internal Medicine, Hyogo College of Medicine

1-1 Mukogawa-cho, Nishinomiya, Hyogo 663-8501, Japan

Tel: +81-798-45-6553; Fax: +81-798-45-6551

E-mail: [okuhara@hyo-med.ac.jp](mailto:okuhara@hyo-med.ac.jp)

Supplementary Table: Characteristics of patients with or without events for 1 year

Absence of events for 1 year (n = 263) Presence of events for 1 year (n = 26)

Age, median (IQR), y 73 (65–78) 78 (67–82) *

Male, n (%) 161 (61.2) 17 (65.4)

NYHA functional class, n (%)

II 242 (92.0) 18 (69.2) *

III 21 (8.0) 8 (30.8) *

Edema, n (%) 83 (31.6) 12 (46.2)

BMI, median (IQR), kg/m2 23.4 (20.7–26.0) 21.5 (19.6–23.5) *

SBP, mean (SD), mm Hg 126 (16) 117 (23) *

HR, median (IQR), beats/min 71 (62–79) 70 (66–79)

Hemoglobin, mean (SD), g/dL 12.8 (1.9) 12.1 (2.2) **

BUN, median (IQR), mg/dL 20 (16–25) 27 (19–37) *

eGFR, median (IQR), mL/min/1.73m2 55.1 (43.1–65.7) 40.9 (29.9–51.1) *

BNP, median (IQR), pg/mL 100 (45–231) 391 (76–939) *

LVEF, median (IQR), % 52 (40–64) 42 (29–64) **

HF type, n (%)

HFrEF 60 (22.8) 12 (46.2) *

HFmrEF 60 (22.8) 2 (7.7)

HFpEF 143 (54.4) 12 (46.2)

LVDd, median (IQR), mm 53.1 (48.0–59.0) 56.0 (48.7–69.0) **

E/e’, median (IQR) 11.9 (8.8–15.7) 16.7 (10.2–23.5) *

Etiology, n (%)

Ischemic etiology 88 (33.5) 8 (30.8)

DCM 59 (22.4) 4 (15.4)

AF or AFL 86 (32.8) 10 (38.5)

Pacemaker implantation 17 (6.5) 6 (23.1) *

Hypertension 168 (63.9) 18 (69.2)

Diabetes mellitus 82 (31.2) 11 (42.3)

Oral medications, n (%)

Loop diuretics 263 (100) 26 (100)

Furosemide equivalent, median (IQR), mg 20 (20–40) 25 (20–45)

Aldosterone antagonists 109 (41.4) 10 (38.5)

ACE-inhibitors or ARBs 191 (72.6) 16 (61.5)

β-blockers 139 (52.9) 16 (61.5)

IQR, interquartile range; SD, standard deviation; NYHA, New York Heart Association; BMI, body mass index; SBP, systolic blood pressure; HR, heart rate; BUN, blood urea nitrogen; eGFR, estimated glomerular filtration rate; BNP, brain natriuretic peptide; LVEF, left ventricular ejection fraction; HF, heart failure; HFrEF, heart failure with reduced ejection fraction; HFmrEF, heart failure with mid-range ejection fraction; HFpEF, heart failure with preserved ejection fraction; LVDd, left ventricular diastolic diameter; DCM, dilated cardiomyopathy; AF, atrial fibrillation; AFL, atrial flutter; ACE, angiotensin-converting enzyme; ARB, angiotensin receptor blocker. * *p* < 0.05, ** *p* < 0.1 vs. absence of events for 1 year

Supplementary Figure: Kaplan–Meier curves for cardiovascular death or unplanned hospital admission according to change in brain natriuretic peptide (BNP)


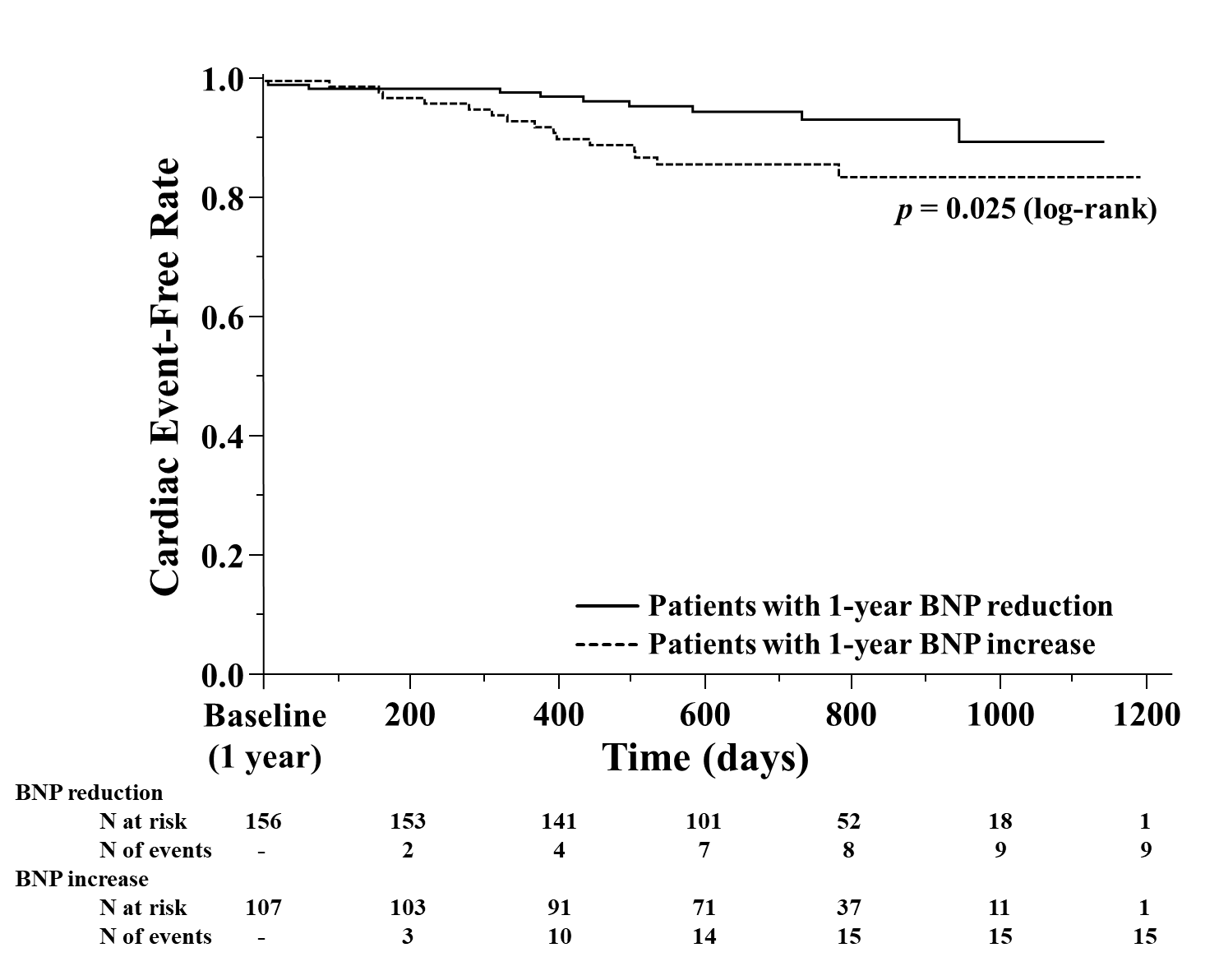

Supplement: Supplementary file 1 — Supplementary Information [file 41598_2019_53697_MOESM1_ESM.docx]
